# Epigallocatechin‐3‐Gallate Encapsulated SNEDDS: A Novel Approach for Neuroprotection in Alzheimer’s Disease

**DOI:** 10.1002/alz70859_102527

**Published:** 2025-12-25

**Authors:** Biswajit Pal, Sachin Kumar Singh, Bushra Bashir, Sukriti Vishwas, Sambita Panda

**Affiliations:** ^1^ Lovely Professional University, Phagwara, Punjab India; ^2^ School of Pharmaceutical Sciences, Lovely Professional University, Jalandhar, Punjab India

## Abstract

**Background:**

Alzheimer's disease (AD) is a progressive neurodegenerative condition associated with cognitive decline and impaired neurocognitive function. It is characterized by neural degeneration, loss of brain tissue, accumulation of amyloid‐beta (Aβ) plaques and neurofibrillary tangles. Even though, several phytochemicals, including fisetin, quercetin, naringenin, berberine, and lycopene, have been studied for their potential in managing AD, epigallocatechin‐3‐gallate (EGCG) stands out for its potent neuroprotective properties, reducing acetylcholinesterase (AChE) activity, Aβ accumulation, oxidative damage, neuroinflammation, and mitochondrial dysfunction. However, EGCG's limited bioavailability and poor blood‐brain barrier penetration hinder its therapeutic efficacy. Self‐emulsifying drug delivery system (SNEDDS) provides several advantages, including enhanced drug solubility, ease of preparation, robust stability, improved bioavailability, and better permeability across the blood‐brain barrier.

**Method:**

To address these limitations, a SNEDDS was developed for EGCG. Further pharmacodynamic studies were conducted using Morris water maize to evaluate the cognition of rats. Moreover, biochemical estimation such as AChE activity, Aβ levels and inflammatory mediators such as TNF‐α, IL‐6 and IL‐8 levels were also assessed.

**Result:**

The optimized EGCG‐loaded SNEDDS formulation demonstrated key characteristics, such as a droplet size of 69.83 nm, a polydispersity index of 0.21, a zeta potential of ‐17 mV and a drug loading efficiency of 96%. Pharmacodynamic evaluations, including tests of cognitive and motor function in rats, showed marked cognitive improvement at both low and high doses. Additionally, biochemical analyses revealed that the EGCG‐loaded SNEDDS significantly reduced AChE activity, Aβ levels, oxidative stress, and neuroinflammatory markers.

**Conclusion:**

EGCG‐loaded SNEDDS served as a therapeutic approach in the management of AD. Therefore, our study suggests a potential neuroprotective strategy of EGCG‐loaded SNEDDS with its beneficial effects of AD.

